# Flexible thin-film black gold membranes with ultrabroadband plasmonic nanofocusing for efficient solar vapour generation

**DOI:** 10.1038/ncomms10103

**Published:** 2015-12-14

**Authors:** Kyuyoung Bae, Gumin Kang, Suehyun K. Cho, Wounjhang Park, Kyoungsik Kim, Willie J. Padilla

**Affiliations:** 1 School of Mechanical Engineering, Yonsei University, 50 Yonsei-ro, Seodaemun-gu, Seoul 120-749, Republic of Korea; 2 Department of Electrical, Computer & Energy Engineering and Materials Science & Engineering Program, University of Colorado, Boulder, Colorado 80309, USA; 3 Department of Electrical and Computer Engineering, Duke University, Durham, North Carolina 27708, USA

## Abstract

Solar steam generation has been achieved by surface plasmon heating with metallic nanoshells or nanoparticles, which have inherently narrow absorption bandwidth. For efficient light-to-heat conversion from a wider solar spectrum, we employ adiabatic plasmonic nanofocusing to attain both polarization-independent ultrabroadband light absorption and high plasmon dissipation loss. Here we demonstrate large area, flexible thin-film black gold membranes, which have multiscale structures of varying metallic nanoscale gaps (0–200 nm) as well as microscale funnel structures. The adiabatic nanofocusing of self-aggregated metallic nanowire bundle arrays produces average absorption of 91% at 400–2,500 nm and the microscale funnel structures lead to average reflection of 7% at 2.5–17 μm. This membrane allows heat localization within the few micrometre-thick layer and continuous water provision through micropores. We efficiently generate water vapour with solar thermal conversion efficiency up to 57% at 20 kW m^−2^. This new structure has a variety of applications in solar energy harvesting, thermoplasmonics and related technologies.

## Abstract (web summary)

Efficient steam generation under solar irradiation is of interest for energy harvesting applications. Here, Bae *et al.* develop a plasmonic nanofocusing film consisting of metal coated alumina nanowires to efficiently generate solar vapour with an efficiency up to 57% at 20 kWm^−2^.

## Graphical abstract

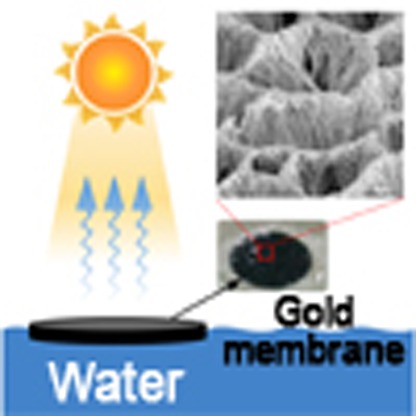

Efficient solar energy harvesting for steam generation has a wide range of applications from small-scale stand-alone solar energy converter for water purification or sterilization systems to large-scale electric power plants and desalination systems^1^,^2^,^3^,^4^. An enhancement of vapour generation efficiency from the sunlight can improve the overall system performances. Several factors have been considered to facilitate efficient steam generation under solar irradiation without any artificial light concentration or electric power: sufficient absorption in the solar spectrum, thermal insulation from heating of bulk water and efficient escaping process of generated vapour bubbles through fluid with minimal heat losses^5^,^6^,^7^,^8^.

The antireflective and light trapping properties of metallic nanoshells or nanoparticles have been used to realize the localized resonant surface plasmon heating, resulting in high light-to-heat conversion efficiencies^5^,^6^,^7^,^8^,^9^,^10^,^11^. Efficient resonant light absorption has been established by metallic gap resonators^12^,^13^, gratings^14^ and microcavities^15^ with surface plasmon by designing appropriate geometries. However, the structures have inherently narrow absorption bandwidth due to the resonant characteristics of surface plasmon excitations. In addition, angle, polarization and wavelength dependence of the plasmonic structures limit the realization of omnidirectional broadband absorber. In contrast, nonresonant broadband absorption of unpolarized light has been demonstrated by touching subwavelength-sized metal cylinders^16^ and spheres^17^. However, the nonresonant absorption remains less efficient at longer wavelengths than at the surface plasmon resonance wavelength.

To attain photothermal conversion using the full solar spectrum from visible to infrared wavelength ranges, adiabatic nanofocusing of surface plasmons is recommended for highly efficient broadband light absorptions, as it excites surface plasmon modes throughout the arrays of ultrasharp metal grooves or subwavelength-sized wedges^18^,^19^,^20^,^21^,^22^,^23^,^24^,^25^. In general, heat generation by plasmonic nanofocusing has long been regarded only as a side effect that had to be avoided, for example, for use in plasmonic waveguide and high-resolution near-field microscopy^26^,^27^,^28^,^29^. Therefore, nanofocusing structures have been optimized to minimize the dissipative loss while maintaining low surface plasmon reflection^30^,^31^,^32^,^33^,^34^. As the structure's taper angle gets smaller, the plasmon reflection decreases while power dissipation loss significantly increases, consequently decreasing plasmon propagation length. Thus, to this day, many efforts have been made to find the optimal taper angle to lower the plasmon reflections and dissipation loss^18^,^20^,^30^,^31^,^32^,^33^,^34^.

In contrast to the competing relationship between plasmon reflection and dissipation loss^20^,^35^, efficient solar steam generation simultaneously requires high surface plasmon dissipation losses for heat generation and the high light absorption in the metal structure. For this purpose, adiabatic nanofocusing of plasmons with the smallest taper angles is perfectly suited. At sufficiently small taper angles near zero, the gap surface plasmon wave vector does not change significantly on the scale of the light wavelength throughout tapered metallic grooves and tips, leading to adiabatic nanofocusing. The plasmon does not experience noticeable reflections and propagates towards the groove bottom or tips, where it asymptotically stops, eventually dissipating in the metal^30^,^36^.

The adiabatic nanofocusing structures with small taper angles, which have high surface plasmon dissipation loss and absorption, are difficult to fabricate for large area because fabrication parameters should be controlled precisely over the whole process. We use a bottom-up self-assembly approach that combines anodization, wet etching and sputtering, which are well established for large-scale fabrication procedures^37^,^38^. Using this technique, we propose a novel concept of self-aggregated metallic nanowire bundle array structure on the microporous substrate to produce flexible thin-film black gold membranes. Our multiscale structure has sufficiently small taper angle (∼1°), hence there is a wide range of metallic nanoscale gaps from zero to hundreds of nanometres over few microns depth as well as 3 μm funnel-shaped structures. The small taper angle and varying nanogaps between aggregated metallic nanowires are responsible for the broadband absorption in the visible to near-infrared region. The 3-μm funnel structure yields ultrabroadband absorption up to 17 μm in the mid-infrared region. Our thin-film black gold membrane absorbs the light with the measured average absorption of 91% in the wavelength range from 400 to 2,500 nm and the average reflection of 7% from 2.5 to 17 μm. Using this film, we efficiently generate solar vapour with solar thermal conversion efficiency up to 57% at an incident intensity of 20 kW m^−2^.

In addition, the thin black gold membrane floats on the water surface and this minimizes the thermal energy loss through the non-vapourizing bulk water because vapour bubbles are able to escape directly from the surface. If the vapourizing hot regions are not appropriately shallow, the vapourized bubbles easily liquefy back while they travel through the water. Our nanofocusing membrane allows for heat localization within the plane of few microns thickness, as well as the continuous water provision from bulk water through micropores in the substrate. Such characteristics allow more efficient solar vapour generation even in lower solar illumination in comparison with nanoparticles^5^,^6^,^7^,^8^,^9^,^10^,^11^.

Here we demonstrate flexible thin-film black gold membranes with multiscale structures of microscale funnel structures and varying metallic nanoscale gaps at the same time. The adiabatic nanofocusing generated by self-aggregated gold-coated nanowire bundles provides high absorption from ultraviolet to infrared regime. The microscale funnel structures also produce high absorption up to 17 μm. This membrane, which floats on the water surface, allows continuous water provision through micropores and heat localization within the few micrometre-thick layer by minimizing the thermal energy loss through the non-vapourizing bulk water. We achieved efficient steam generation with solar thermal conversion efficiency up to 57% at 20 kW m^−2^.

## Results

### Fabrication of black gold membranes

**Figure 1:**
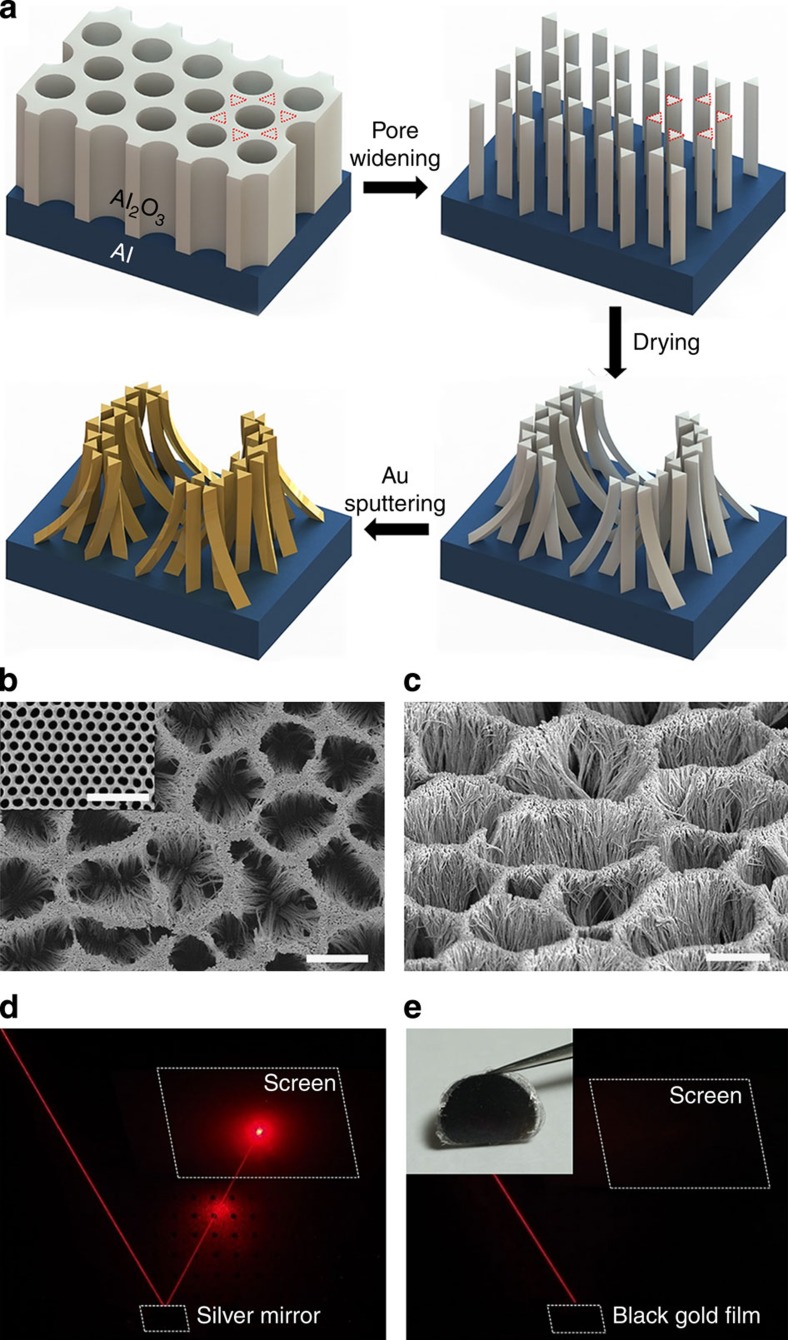
Schematics and structure of a thin-film black gold membrane. (**a**) Schematic illustration of the method to fabricate black gold membranes by employing pore-widening process into hexagonal AAO templates. (**b**,**c**) SEM images of black gold membrane (**b**) with top view, (**c**) with bird's eye view (52° tilted) and ordered hexagonal array of AAO template (**b**, inset). Scale bars, 2 μm (**b**,**c**) and 500 nm (inset), respectively. (**d**,**e**) Optical photographs displaying the laser beam path reflected by (**d**) a silver mirror and (**e**) a black gold membrane. Inset of **e** is the photo of a membrane.

As shown in Fig. 1a, we fabricated a flexible thin-film membrane by self-aggregation of collapsed nanowires after appropriate pore widening of the anodic aluminium oxide (AAO) templates prepared by a conventional two-step anodization process^39^, which offers precisely ordered hexagonal nanopore arrays (the inset of Fig. 1b, see Methods section). By employing pore-widening processes with controlled wet etching time into AAO templates, we get self-aggregated nanowire bundles. To widen the nanoholes of porous AAO templates with initial pore size of 30 nm, we used chemical wet etching with 5 wt% phosphoric acid. The etching time and temperature dominantly determine the enlarged diameter of the porous AAO nanoholes. At a fixed temperature of 30 °C, we controlled the etching time in a range of 0–50 min. As we enlarged the diameter of nanoholes, surrounded by thin alumina nanowalls, the neighbouring pores began to merge after the etching time of ∼42 min. After a suitable etching time of about ∼45 min, the appropriate conglomerations between the nearest neighbouring pores allowed the residuals of alumina nanowalls to become vertically aligned nanowire arrays resembling triangular-shaped cross sections. To terminate the pore-widening, we took the AAO sample out and subsequently washed with water before drying in air. In the drying process, if the water–air surface tension between two alumina nanowires exceeds the stiffness of the nanowire, then the nanowires will bend towards the surrounding nanowires. If this bending force is greater than the yield strength, the bending nanowires will plastically collapse, resulting in the formation of self-aggregated nanowire bundles^40^. Because the surface tensions are in random directions, our self-aggregated nanowire bundles displayed irregular three-dimensional (3D) patterns similar to mountain ridges and valleys, as shown in the scanning electron microscopy (SEM) images of Fig. 1b,c. On top of the structure, we sputtered gold film with 40 nm thickness and the film appeared black in colour. Then, we peeled off the gold-coated film with a scotch tape. The optical photograph of the flexible sample is shown in the inset of Fig. 1e. To test the blackness of our sample, He–Ne laser light with a wavelength of 633 nm was incident on silver mirror and the film, respectively, and the incident and reflected beam path of light are shown in Fig. 1d,e. The specular reflectance of our sample is significantly smaller than that of a silver mirror and the detailed explanation will be presented later.

### Structure modelling for numerical simulations

**Figure 2:**
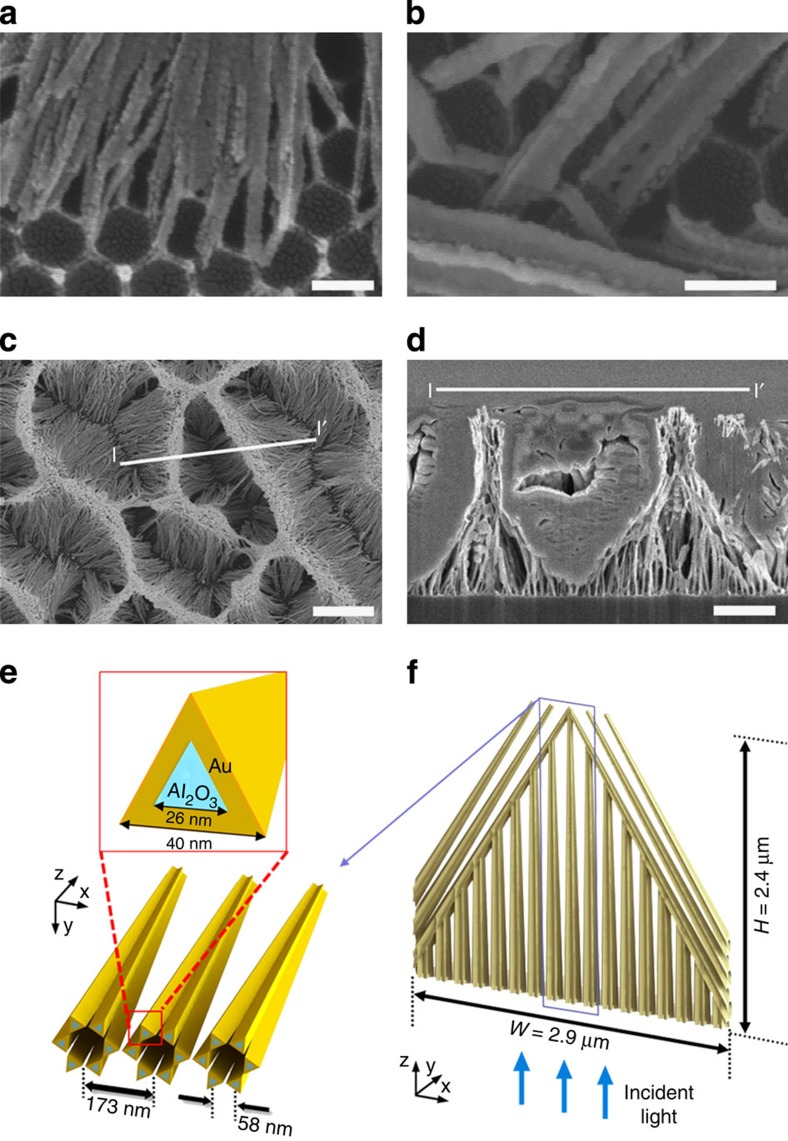
Theoretical modelling. (**a**–**d**) SEM images of our structure are presented. (**a**) Collapsed alumina nanowires due to the capillary-force in drying process are linked to the vertices of hexagonal unit at bottom. (**b**) After gold coating, the sizes of nanowires are measured. (**c**) The top ends of collapsed nanowires conglomerate to form ridges. (**d**) Cross-sectional SEM image of black gold membrane presents funnel-shaped bundles. Scale bars, (**a**,**b**) 100 nm (**c**) 2 μm and (**d**) 1 μm, respectively. (**e**,**f**) Theoretical modelling of the self-aggregated structure. (**e**) Star of David shaped hexagonal unit of nanowires with concentric triangular cross sections of gold coating on alumina (top) merges at the top end. (**f**) A funnel-shaped bundle is modelled by aligning merging hexagonal units as vertical or slanted.

For quantitative analysis, firstly we measured the shapes and sizes of collapsed alumina nanowires before gold coating. The plastically collapsed alumina nanowires, induced by surface tension in drying process, are shown in the SEM image of Fig. 2a. Even after the collapse, the bottom ends of nanowires were mechanically connected into each vertex of hexagonal units of the aluminium substrate that were formed during anodization process. The side length of the hexagonal unit cell is 58 nm. We also observed that the alumina nanowire has triangular cross section with side length of 26 nm. After the gold coating of nanowires with 40 nm thickness, as presented in Fig. 2b, the side length of the triangular cross section of metallic nanowire was 40 nm. The experimentally obtained images show that thin gold films are surrounding an alumina nanowire with equilateral triangular cross sections with side lengths of 26 and 40 nm for the alumina and surrounding gold-coated regions, respectively, as shown in Fig. 2e.

As previously mentioned, our self-aggregated metallic nanowire bundles have random 3D patterns that resemble mountain ridges and valleys, as displayed in Fig. 2c. To make a faithful model of this rather complex structure with reasonable simplifications, we constructed our model with two unit structures at different scales. First, we note that the top ends of nanowires conglomerate to form a ridge, while the bottom ends are fixed to the vertices of hexagonal units. To model this six collapsed nanowires linked to a unit hexagon at bottom substrate, we introduced a ‘merged nanowire hexagonal unit' as demonstrated in Fig. 2e. All gold-coated nanowires were fixed at the vertices of a 58 nm side-length hexagon at the bottom end, yet they bound together at the top end. The nanowires merged at the top ridge with a high packing density, hence we can conclude they are completely conglomerated. Six gold-coated alumina nanowires with triangular cross section were assembled in the shape of the Star of David with 11 nm gaps between the closest triangles at the bottom ends. These slanted six wires were bound together at the top ends. By defining the rectangular computation domain as 173 nm by 200 nm in width and height with periodic boundary condition in finite-difference time-domain (FDTD) simulations, we can model the hexagonal crystal structure of 100 nm interpore distance.

The cross-sectional focused ion beam SEM image of film membrane through points between l and l′ is presented in Fig. 2d. The image shows that a self-aggregated bundle has the shape of a concave funnel structure with an inclination of 33° as well as dimensions of 2.4 μm height by 2.9 μm width. Because a total of 17 merged nanowire hexagonal units (173 nm lattice constant) exist inside a funnel-shaped bundle, we constructed a larger unit structure of funnel-shaped bundle as plotted in Fig. 2f, comprised of 17 vertical and 6 slanted merged nanowire hexagonal units. To acquire the inclined sides of the funnel structure, we removed materials outside the triangular region with dimensions of 2.4 μm by 2.9 μm. Then, we put the three merged nanowire hexagonal units into a constant slope of 33° on each of the left and right sides, while the other units were vertically aligned. This layer becomes a flexible thin-film black gold membrane after being peeled off from the aluminium substrate. The incident light propagates upward from the bottom of this membrane.

### Ultrabroadband plasmonic nanofocusing effect

**Figure 3:**
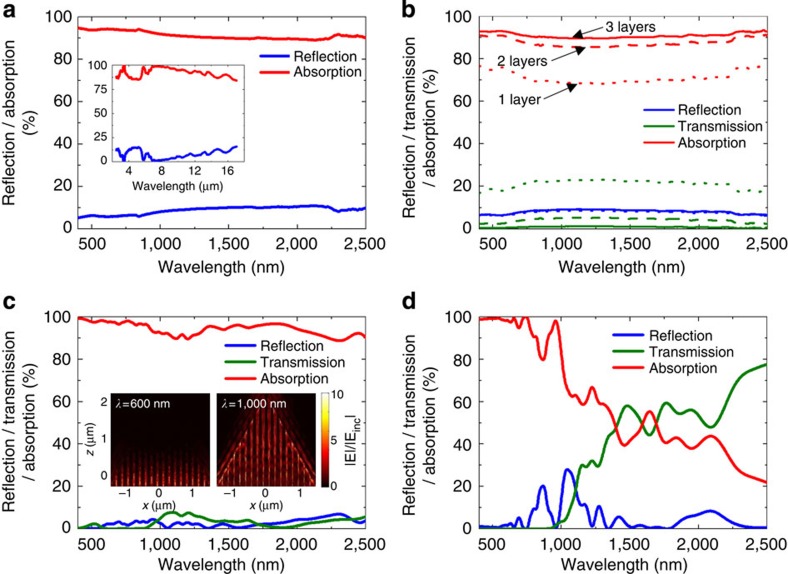
Optical properties of black gold membrane. (**a**) Experimental measurements of optical behaviours of a black gold membrane peeled off with aluminium tape. Total reflection and absorption in the wavelength range of 400–2,500 nm. The inset represents reflection and absorption measured by Fourier transform infrared spectroscopy from 2.5 to 17 μm. (**b**) Optical properties of black gold membrane peeled off with 3M Micropore tape. Dotted, dashed and solid lines represent one layer, two layers and three layers, respectively. As the number of layers increases, the transmission decreases while the reflection is almost the same, resulting in the increase of absorption. (**c**,**d**) Theoretical FDTD calculations with structure modelling. Simulated absorption spectra using (**c**) funnel-shaped bundle structure model and (**d**) a merged Star of David nanowire hexagonal unit. The insets of **c** are the electric field profiles in *x–z* plane for a funnel-shaped bundle at (left) *λ*=600 nm and (right) 1,000 nm.

Figure 3a shows the experimental measurement of total transmission, reflection and absorption spectra for a black gold membrane on Al tape, using a spectrophotometer system (UV3600, Shimadzu Scientific Instruments) with a 60-mm diameter integrating sphere (MPC-3100). In order to characterize the intrinsic optical properties of the black gold membrane structure, we employed the membrane on aluminium tape, which allows near-perfect membrane transfer with virtually no unwanted defects. Specular and diffuse properties are added together in the spectra. The membrane has broadband antireflective property with significantly low total reflection (specular plus diffuse reflection) of less than ∼10% in the range of 400–2,500 nm. Moreover, we also confirmed that our membrane has polarization-independent total reflection, as shown in Supplementary Fig. 1, attained by using a broadband polarizer (Ultrabroadband wire grid polarizer: 250 nm–4 μm, WP25M-UB, Thorlabs). The transmission is nearly nonexistent due to the high reflectance of Al tape. The total average absorption was observed to be as high as 91% in the wide range of 400–2,500 nm. We also took the reflection spectrum with a Fourier transform infrared spectroscopy (FTIR) in the mid-infrared region, as plotted in the inset of Fig. 3a, and the average reflection was as low as ∼7% below the wavelength of 17 μm. The total reflection spectrum measured from an FTIR spectrometer (Nicolet 6700, Thermo Electron Corporation) with an integrating sphere accessory (SPECTRAFIRE) is also presented in Supplementary Fig. 2, which are in good agreement with the inset of Fig. 3a obtained with an FTIR with an objective lens. These results experimentally verify the ultrabroadband high-absorption property of the black gold membrane^41^.

For efficient steam generation, we are going to float the black gold membrane peeled off by microporous substrate (3M Micropore Surgical Tape 1530S-1) on the surface of water. The detailed explanation will be given later. Thereby, the optical properties of black gold membrane transferred on micropore tape are also measured in Fig. 3b for one, two or three stacked membranes (schematic images are described in Supplementary Fig. 3). One membrane on micropore tape has transmission of ∼21%, which arise from the microscale cracks generated due to the porous characteristics of the tape (Supplementary Fig. 4). If we add the second and third layers, so as to reduce optical loss caused by light transmission through the first membrane, the average absorption can be obtained as high as 91%.

To theoretically investigate the optical behaviours of a black gold membrane, we numerically calculated spatial distributions of E-field intensity, transmission and reflection spectra using 3D FDTD simulations (Lumerical Solutions). The normal upward plane-wave light with *x* axis polarization is incident in the structure model of Fig. 2f. We applied the broadband wavelength in the range of 400–2,500 nm. Periodic boundary conditions are applied along the *x*- and *y*-directions and perfectly matched layers in the *z*-direction. We performed 3D simulations with a cubic mesh size of 4 nm.

Figure 3c,d show the FDTD simulated transmission, reflection and absorption spectra for a funnel-shaped bundle and a merged nanowire hexagonal unit, respectively. The simulated absorption of a merged nanowire hexagonal unit is high in the range of 400–1,000 nm, then decreases rapidly at wavelengths longer than 1,000 nm as plotted in Fig. 3d. For the merged nanowire hexagonal unit, the largest gap between neighbouring triangles is 11 nm that linearly decreases to zero along the few micron length of nanowires. Thanks to the Star of David shaped assembly of gold-coated triangles with 152 nm diameter at the bottom surface, a wide range of nano gap distances are present with various taper angles and/or between various gold-coated triangles. Resonant plasmon nanofocusing appears at shorter wavelength in narrow nanogaps while at longer wavelength in larger nanogaps. Because the diameter of a hexagonal unit is at most ∼150 nm, resonant nanofocusing appears in the range up to ∼1 μm but is not as efficient at longer wavelength.

On the other hand, the simulated absorption of a self-aggregated funnel-shaped bundle structure is efficient up to 2,500 nm, as presented in Fig. 3c. Along with the Star of David unit, vertically aligned 17 units with 173 nm distance in between allow us to get a much broader range of gap widths between gold layers by combining nanowires from different hexagonal units (Fig. 2f). This wider range of nanogaps broadens the range of absorption up to the near-infrared region. In addition, the funnel shape, made of gold layer with 2.9 μm lattice, behaves similarly to metallic microcone structure, resulting in a ultrabroadband absorption as far as 17 μm in the mid-infrared. The insets of Fig. 3c show the electric field distribution in the funnel-shaped bundle at the wavelength of 600 nm and 1,000 nm, respectively. They confirm the light coupling into surface plasmon modes and subsquent nanofocusing and also show that the shorter wanvelength light is absorbed mostly by the merged nanowire hexagonal units, while the longer wavelength light is dissipated throughout the entire funnel structure.

The ultrabroadband absorption of our black gold membrane is originated from the multiscale structure of various nanoscale gaps over few microns depth and microscale funnel shape. Metallic nanowires collapsed and then aggregated in arbitrary directions to form the membrane; thereby ridges and valleys are randomly distributed, while the cross sections have common funnel shapes. This inhomogeneity in morphology of the actual sample has an effect of averaging out the narrow absorption peaks in comparison with our simulation result. The extinction, that is, absorption plus scattering, is experimentally observed as >99% in the range of 400–1,000 nm, using the relationship of 1−*R*_spec_−*T*_spec_ (the specular transmission (*T*_spec_) and reflection (*R*_spec_)), as plotted in Supplementary Fig. 5. In addition, the extinction from various incidence angles are plotted in Supplementary Fig. 6.

### Solar steam generation experiments

**Figure 4:**
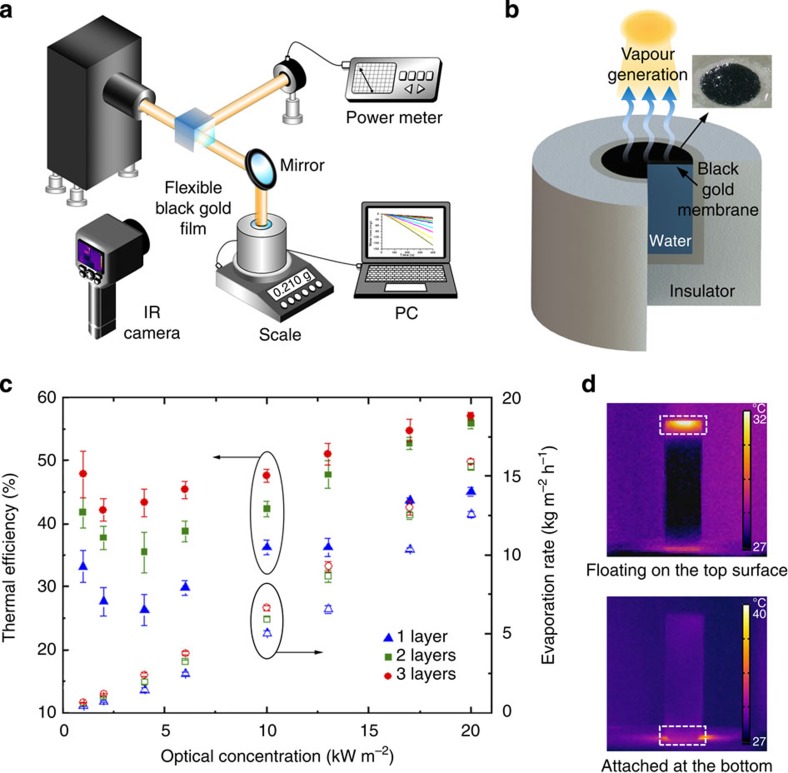
Solar vapour generation with black gold membranes. (**a**) Schematics of experimental setup for solar vapour generation. (**b**) Schematic representation of vapour generation and a membrane sample floating on the water surface (inset). (**c**) The thermal efficiency (solid dot) and evaporation rate (open dot) of vapour generation process generated by one, two or three layers of black gold membranes at varying optical power density. The error bars are defined as the s.d. (**d**) Side-view infrared images of the black gold membrane under the same light illumination of 10 kW m^−2^ when floating on the top surface of the water cuvette (top) and attached at the bottom of the cuvette (bottom). The measurements were through the quartz cuvette walls.

To obtain the light-to-thermal conversion efficiency of our membrane, we measured evaporation rates of water using an experimental setup, as shown in Fig. 4a. The setup is equipped with an illumination source, power metre, test chamber, scale, infrared camera and data collection system. To simulate solar light, we used a xenon arc lamp with air mass filter (AM 1.5G filter, Sciencetech) as an illumination source^42^,^43^,^44^. The test chamber consists of an acrylic tube with inside diameters of 20 mm and height of 45 mm. To minimize two-dimensional thermal losses in the test chamber, we surrounded the acrylic tube with styrofoam, then put the chamber on the scale as represented in Fig. 4b. We floated the black gold membrane peeled off by microporous substrate (3M Micropore Surgical Tape 1530S-1) on the surface of water in the tube. We used this tape because its interconnected micropores provide hydrophilicity, thus encouraging underlying fluid flow to the top surface through the tape. 3M Micropore tape is initially hydrophobic in the air, as shown in Supplementary Fig. 7, hence the contact angle of a water drop is 126°. Many open micropores contain air cavities that increase the air–water interfaces area, which makes the surface hydrophobic. However, after we soak this tape into water, the wet tape's contact angle decreases to 26°, which represents hydrophilicity. For the sufficiently soaked tape, the air inside micropores escapes and water completely fills the micropores that attract water by capillary forces. This leads to hydrophilicity and finally provides the paths for fluid to flow to the surface through the pores. The water flow through the micropores enables continuous solar vapour generation with constant water provision. Furthermore, 3M Micropore tape has low enough mass density to float on the surface of water (see the inset of Fig. 4b).

The evaporation rates of water with black gold film are measured by mass change as a function of time under varying light illumination from 1 to 20 kW m^−2^. The photograph of vapour generation by the film is presented in Supplementary Fig. 8. At each optical concentration, we measured mass changes during 900 s and evaporation rate was mostly constant after specific initial transient period. The water evaporation rate in the ambient state without illumination was 1.13 kg m^−2^ h^−1^. This ambient evaporation rate was subtracted from all evaporation rates under the solar illuminations to investigate the vapour generation only from solar illumination.

We measured the mass loss of water with 1, 2 or 3 membrane layers and without membrane at various optical concentrations (Supplementary Figs 9 and 10). The evaporation rate increased with increasing illumination intensity. For the single layer, the evaporation rates were 0.47 and 12.60 kg m^−2^ h^−1^ for light intensities of 1 and 20 kW m^−2^, respectively. We presented the optical properties of black gold membrane transferred on micropore tape in Fig. 3b for one, two or three stacked membranes. Due to the microscale cracks generated by the porous characteristics of the tape, a membrane on micropore tape has transmission of ∼21% (see the crack image in Supplementary Fig. 4a). Even though the cracks results in light leakage through the black gold membrane, the micropores are important for efficient solar steam generation because they allow water to flow through to continue the evaporation process. Hence, we added the second or third layer so as to reduce optical loss caused by light transmission through the first membrane and also to lower the thermal loss into underlying water. As a result, for two (or three) layers, the evaporation rates are enhanced to 0.58 (or 0.67) and 15.62 (or 15.95) kg m^−2^ h^−1^ for intensities of 1 and 20 kW m^−2^, respectively.

The evaporation rate as a function of power density is shown in Fig. 4c. As the solar energy increases, the evaporation rate increases. The thermal receiver efficiency (*η*_th_) is applied to evaluate the efficiency of black gold membrane and is defined as


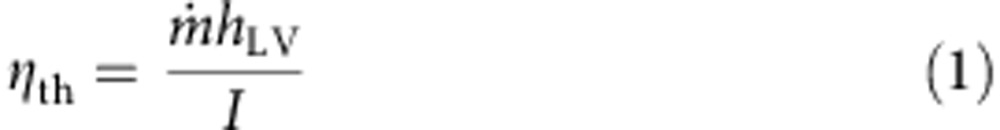


where 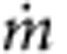 is mass flux, *h*_LV_ is total enthalpy of sensible heat and liquid–vapour phase change and *I* is the power density of light illumination^8^. The thermal efficiency *η*_th_ of a black gold membrane is in the range of 26–45%. For the single layer, solar energy conversion to heat for the vapour generation is limited by thermal and optical losses. The *η*_th_ increases to the range of 36 to 56% for two layers and 42–57% for three layers. In these results, the additional layers recycle the transmitted light through the first membrane and reduce the thermal losses through underlying water.

One of the bottlenecks for efficient solar vapour generation is that vapourized gas may liquefy back while the vapour bubbles travel through the water region, if the vapourizing hot spots are not sufficiently shallow for vapour to easily escape in the air before liquefaction. Our floating membrane offers localized hot zones near the water surface and it enables efficient evaporation of the very thin water layer on top of the membrane. The thin-film membrane allows nanofocusing heat localization within the layer of few microns thickness. When this membrane keeps appropriate position from the top surface of water, while receiving continuous water provision through the micropores of the tape, heat localization by nanofocusing enables us to achieve efficient solar vapour generation even with lower solar illumination. Figure 4d is the thermal images by infrared camera (E6, FLIR Systems, Inc.) when the black gold membrane is floating on the top surface of the water cuvette (top) and attached at the bottom of the 45-mm height cuvette (bottom) under the same solar illumination of 10 kW m^−2^. In both cases, we experimentally measured temperature changes at the water surface and 15 mm below the surface, as plotted in Supplementary Fig. 11. We note that temperature values could have small difference due to the infrared absorption by the sidewalls of quartz cuvettes. Nevertheless, the relative temperature distribution at different locations of the cuvette provides meaningful information for qualitative comparison. When the black gold membrane is floating on the surface, the temperature rises only on the surface up to Δ*T*=40 °C, while at the depth of 15 mm the temperature increases only by Δ*T*=9 °C. If we attach a membrane at the bottom of the cuvette, the temperatures at surface and at 15 mm below the surface rise similarly by Δ*T*=14 °C. These results confirm that the hot zones are strongly localized on the surface of water with a floating membrane, resulting in a higher evaporation rate. This heat localization, provided by a floating membrane, leads to a significant reduction of thermal losses through the bulk fluid and consequently an enhancement of the solar thermal conversion efficiency.

**Figure 5:**
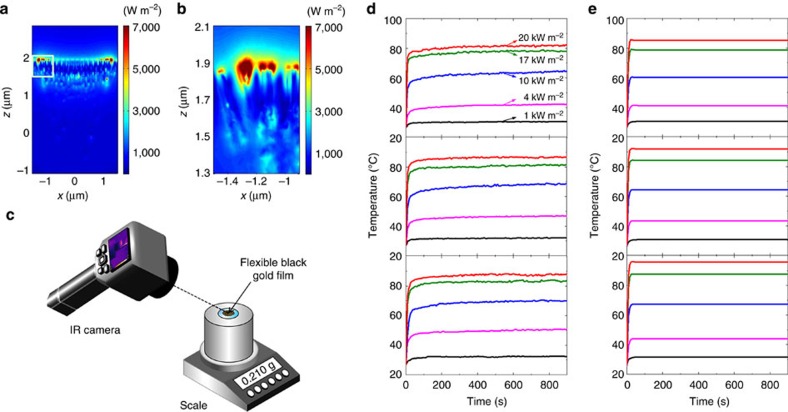
Thermal responses of the black gold membranes. (**a**) Simulated heat flux profile around the black gold membrane illuminated with 20 kW m^−2^. (**b**) Enlarged heat flux profile for the region indicated by a box in **a**. (**c**) Schematic image of water surface temperatures measurement. (**d**) Experimentally measured water temperature as a function of time for one-layer (top), two-layer (middle) and three-layer (bottom) black gold membranes. (**e**) Simulated water temperature as a function of time for one-layer (top), two-layer (middle) and three-layer (bottom) black gold membranes.

To model the heating of water by black gold, we conducted numerical simulations by using the commercial software COMSOL. In order to model the nanoscale heating by the gold nanostructures within the black gold membrane, the heat source is defined by using the FDTD simulations described earlier. The power absorbed by the black gold membrane can be calculated from the Poynting vector theorem. Because our sample materials are non-magnetic, absorbed power can be calculated simply by


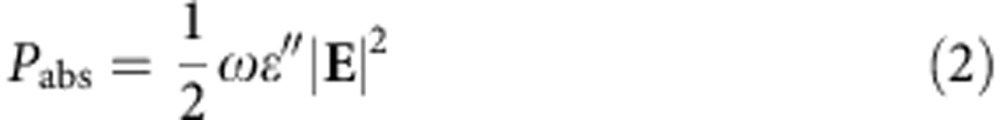


where *ω* is the frequency, *ɛ*′′ is the imaginary part of the dielectric permittivity and **E** is the total electric field^13^. Electric fields are calculated within the 3D region that covers all the structures (Fig. 2f), and absorbed power integrated over the wavelength range of 400–2,500 nm and then imported into COMSOL as nanostructured heat source. To precisely model the nanoscale profile of the heat source, we use a mesh size of 4 nm for regions where the hot spots are formed by the nanofocusing effect and gradually larger mesh sizes for other regions. Mimicking the experimental setup, the computation cell contains 10-μm thick water above the black gold membranes (Supplementary Fig. 12a). The simulation results show that the heat quickly dissipates through water, and the entire volume of water above the black gold membranes is heated uniformly with only very slightly increased temperature gradient near the hot spots. As shown in Fig. 5a,b, the heat flux becomes high near the hot spots of the source profile, reaching ∼7,000 W m^−2^. The heat generated by the hot spots quickly dissipates into the surrounding volume of water and the temperature remains constant throughout the water volume. The simulations of heat flux with other cross sections are also presented in Supplementary Fig. 12. The water temperature is calculated as a function of time and compared with the temperature measured by the thermal imaging infrared camera. To get the temperature dynamics of surface water, we carefully measured the temperature with infrared camera directly through the air, not through the cuvette wall as shown in Fig. 5c–e, show the experimental and simulation results for temperature change of water above one-, two- and three-layer black gold membranes, respectively. Temperatures rise quickly and reach a nearly steady state after 30 s, and the temperatures in the steady state increase as the solar irradiance is increased. The simulated steady state temperatures agree well with the experimental values with errors of only a few degrees.

To assess the thermoplasmonic performance, we consider the integrated absorption efficiency, which is defined as the ratio of total absorbed power to the total incident power integrated over the entire range of wavelength of interest. For our structure, the total absorbed power integrated from 400 to 2,500 nm is 5.00 × 10^−10^ W over the cross-sectional area of 4.45 × 10^−13^ m^2^. The integrated incident power of 1 kW m^−2^ solar spectrum over the same cross-sectional area is 4.01 × 10^−10^ W, yielding an absorption efficiency of 125%. For comparison's sake, we also consider the integrated absorption efficiency of gold nanoshell. Thanks to the spherical symmetry of nanoshell, the absorption cross section of a nanoshell can be calculated analytically by the Mie theory^11^. The integrated absorption efficiency is then given as,


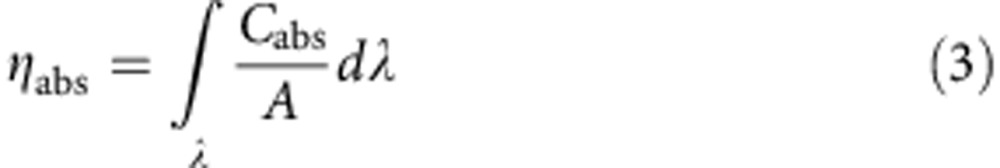


where *C*_abs_ is absorption cross section and *A* is the physical cross section. For a gold nanoshell with core radius of 60 nm and shell thickness of 13 nm (Supplementary Fig. 13), the total absorbed power integrated from 250 nm to 4 μm is 1.49 × 10^−11^ W and the physical cross section is 1.67 × 10^−14^ m^2^, yielding an integrated absorption efficiency of 99%. Note that this comparison is made for a single nanoshell versus a unit structure of our black gold membrane. The total heating effect will obviously depend on the density of these unit structures in the system. Yet, it is nevertheless useful to compare the performance of these unit structures. In both cases, high absorption efficiency is achieved by plasmon resonance. However, our black gold membrane shows a higher integrated absorption efficiency thanks to the broadband spectral response.

## Discussion

Efficient steam generations under solar irradiation have been realized with metallic nanoshells or nanoparticles, which have inherently narrow absorption bandwidth induced by resonant surface plasmon heating. We propose adiabatic nanofocusing structures of surface plasmons, which offer both polarization-independent ultrabroadband light absorption and high plasmon dissipation loss, for attaining highly efficient light-to-heat conversion devices. By employing a large-scale self-assembly approach, we demonstrated flexible thin-film black gold membranes that realize adiabatic nanofocusing of surface plasmons. After AAO templates underwent appropriate pore-widening processes, we got plastically collapsed alumina nanowires with sufficiently small taper angle (∼1°), induced by surface tension in drying process, and then gold coated. Our self-aggregated metallic nanowire bundles display random 3D patterns similar to mountain ridges and valleys, which have the cross sections of microscale funnel shapes. The bundles have multiscale structure with a significantly wide range of metallic nanoscale gaps from zero to hundreds of nanometres over few microns depth and microscale funnel structures, leading to the ultrabroadband absorption of the membrane. The small taper angle and varying nanogaps between aggregated nanowires are responsible for the broadband absorption in the visible to near-infrared region. The 3-μm funnel structure yields the ultrabroadband absorption up to 17 μm in the mid-infrared region. This film had an average absorption of 91% in the wavelength range from 400 to 2,500 nm and the average reflection of 7% from 2.5 to 17 μm. Using this film, we efficiently generated solar vapour with solar thermal conversion efficiency up to 57% at light illumination of 20 kW m^−2^. This few micrometer-thick membrane was attached on a micropore tape that floated on the water surface. On the membrane, the vapourized bubbles escape directly into air, while hydrophilicity of the membrane continuously provides underlying water to the surface through the micropores. If the vapourizing hot spots are inefficiently shallow, the vapourized bubbles easily liquefy back while they travel through the water. The heat localization on the surface minimizes the thermal energy losses into bulk water, and thus enhances the efficiency of solar vapour generation in comparison with dispersed nanoparticles^5^,^6^,^7^. This ultrabroadband absorber membrane in the visible to mid-infrared region opens new approaches for solar energy harvesting and thermoplasmonics applications.

## Methods

### Preparation of hexagonal AAO templates

High-purity aluminium foil (99.999%) with a thickness of 0.5 mm was electropolished in a mixture of perchloric acid and ethanol (HClO_4_:C_2_H_5_OH=1:4 in volumetric ratio) at 20 V. The first anodization was carried out under a constant voltage of 40 V in 0.3 M oxalic acid solution at 10 °C, so that we could get a hexagonal array with an interpore distance of 100 nm. After the first anodization, the AAO layer was removed in a mixture of 1.8 wt% chromic acid and 6 wt% phosphoric acid at 80 °C. After the AAO layer was completely etched, the second anodization was carried out under the same condition for 1 h, resulting in 5-μm thick well-ordered hexagonal AAO template.

### Optical measurements

To characterize the total spectral absorptions of our samples, we carried out absolute hemispherical measurements using a ultraviolet–visible–near-infrared spectrophotometer system (UV3600, Shimadzu Scientific Instruments) with a 60-mm diameter integrating sphere (MPC-3100) by scanning a monochromator coupled to a halogen lamp. The reflected (transmitted) beam including both specular and diffuse reflections (transmissions) from the sample were scattered and collected in an integrating sphere and measured using a photomultiplier tube detector. The reflection was normalized to a BaSO_4_ reference. From Kirchhoff's law, the sample's total absorption can be obtained as *A*=1−(*T*+*R*), using the measured total reflection/transmission, including both specular and diffuse reflections/transmissions. Infrared spectra were collected by FTIR spectrometer (Bruker IFS-66/S), which combined with a × 15 cassegrain objective lens (NA=0.58). Spectra were acquired in the 4,000–600 cm^−1^ range with a resolution of 0.1 cm^−1^ and calibrated with a gold mirror reference (ref. 41).

### FDTD simulations

We performed numerical simulation using Lumerical Solutions, a commercial FDTD simulation software package. The unit cell of a funnel-shaped nanowire bundle structure was designed from the SEM image data of the membrane. Periodic boundary conditions were applied along the *x* and *y* axes, and perfectly matched layers along the *z* axis. Electric fields and absorbed power were detected within the advanced power absorbed monitor of the optical power analysis. The simulations were performed in 3D layout, and a cubic mesh size of 4 nm was employed for the structure region. Optical properties of Au and Al_2_O_3_ were taken from Johnson and Christy^45^ and Palik's Handbook of Optical Constants^46^, respectively, in the spectrum range from 400 to 2,500 nm.

## Additional information

**How to cite this article:** Bae, K. *et al.* Flexible thin-film black gold membranes with ultrabroadband plasmonic nanofocusing for efficient solar vapour generation. *Nat. Commun.* 6:10103 doi: 10.1038/ncomms10103 (2015).

## Supplementary Material

Supplementary Information Supplementary Figures 1-13.
