# Supplementary material for: Flexible thin-film black gold membranes with ultrabroadband plasmonic nanofocusing for efficient solar vapour generation
Source: Nat Commun. 2015 Dec 14;6:10103. doi: 10.1038/ncomms10103 (PMC4682046; doi:10.1038/ncomms10103)
Supplement: Supplementary Information — Supplementary Figures 1-13. [file ncomms10103-s1.pdf]

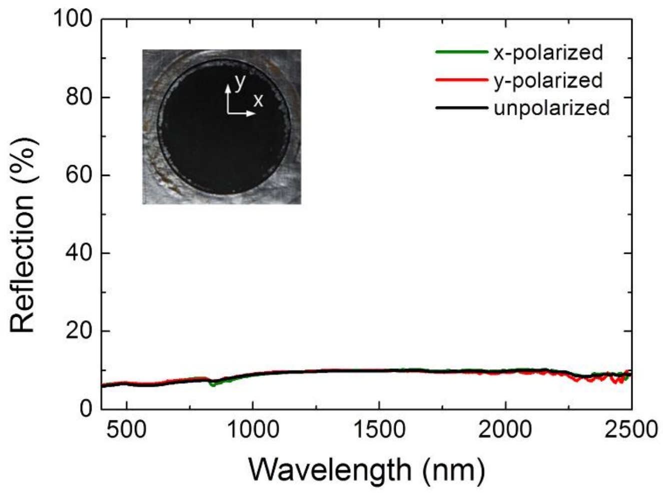

Supplementary Figure 1 | Measured total reflection spectra of x- and y-polarized light. The reflection of black gold membrane peeled off by aluminum tape measured by a UV–vis–NIR spectrophotometer system (UV3600, Shimadzu Scientific Instruments) with a 60 mm-diameter integrating sphere (MPC-3100).

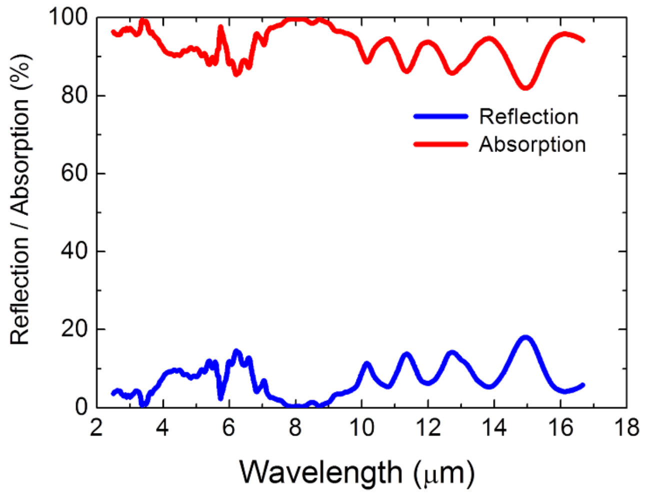

Supplementary Figure 2 | Total infrared reflection and absorption spectra of black gold film on the aluminum tape. The hemispherical (total) reflection is

measured from an FTIR spectrometer (Nicolet 6700, Thermo Electron Corporation) with an integrating sphere accessory (SPECTRAFIRE) from 2.5 μm to 17 μm.

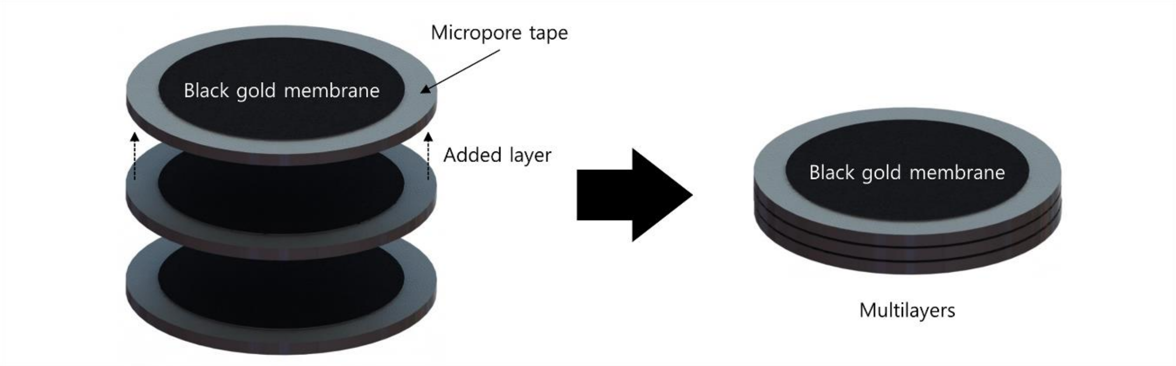

Supplementary Figure 3 | Schematic image of multilayer black gold membrane. The black gold nanostructures are on the 3M micropore tapes, and they are stacked up for 2 and 3layers.

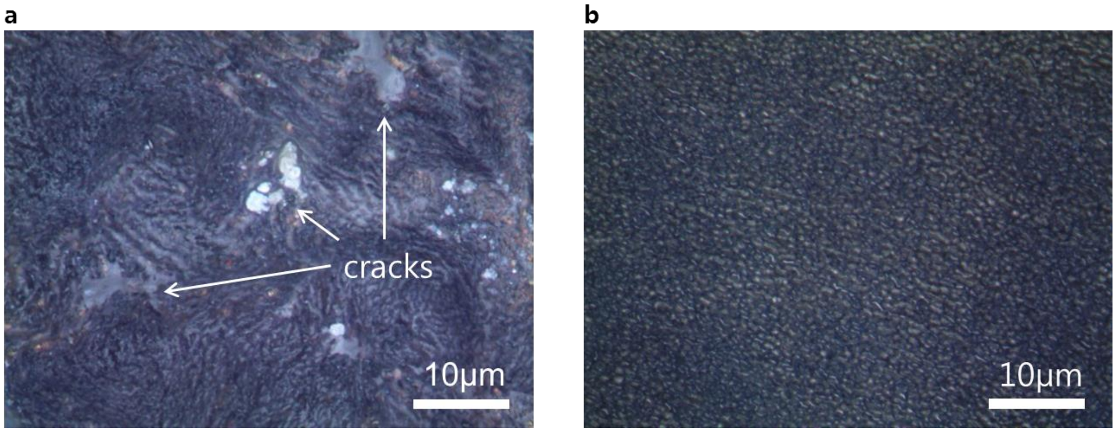

Supplementary Figure 4 | Microscope images of black gold membrane. The membranes were peeled off with (a) 3M Micropore tape and (b) aluminum tape. Microscale cracks appear in black gold membrane peeled off by 3M Micropore tape in contrast to the almost crack-free membrane on aluminum tape.

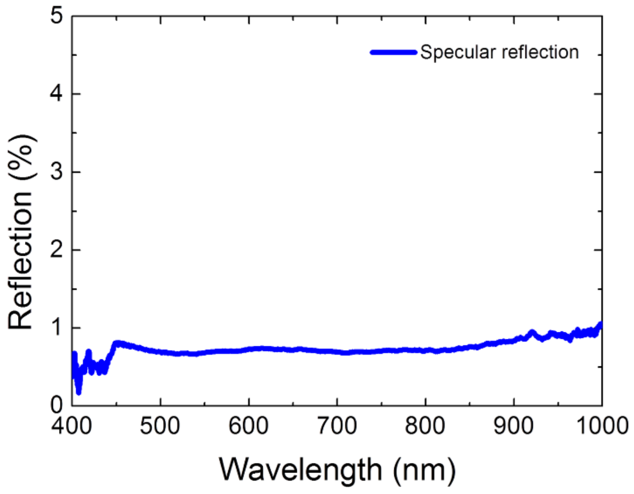

Supplementary Figure 5 | Measured optical properties of black gold membrane peeled off by Aluminum tape. Specular reflection spectrum which was measured using spatially resolved linear reflection spectroscopy. The set-up includes a fibre-coupled grating spectrometer (Ocean optics USB2000+) and Tungsten Halogen light source. We used a reference silver mirror as a calibration reflection measurement. The inset is the optical image of black gold membrane peeled off by aluminum tape. Since the transmission is zero due to Al back reflector, the extinction can be defined by 1- R_{spec} (specular reflection). The average reflection of the film is 0.7%, and the average extinction is 99%.

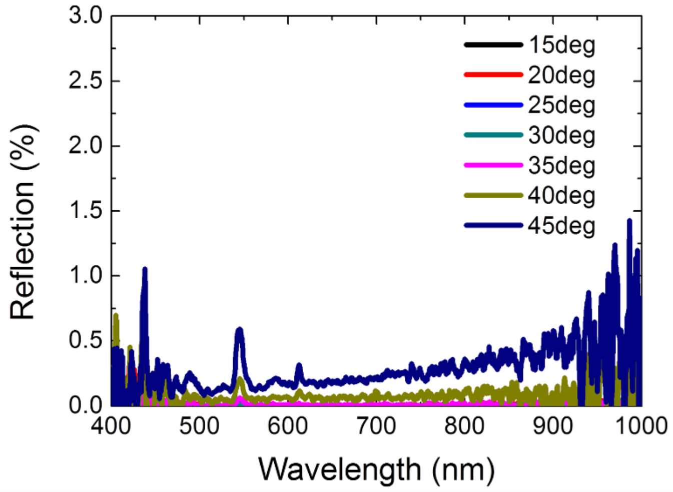

Supplementary Figure 6 | Measured spectra with various incident angles. All spectra were calibrated by a reference silver mirror. To measure various angle, we used various angle reflection sampling system (RSS-VA, Ocean optics Inc.)

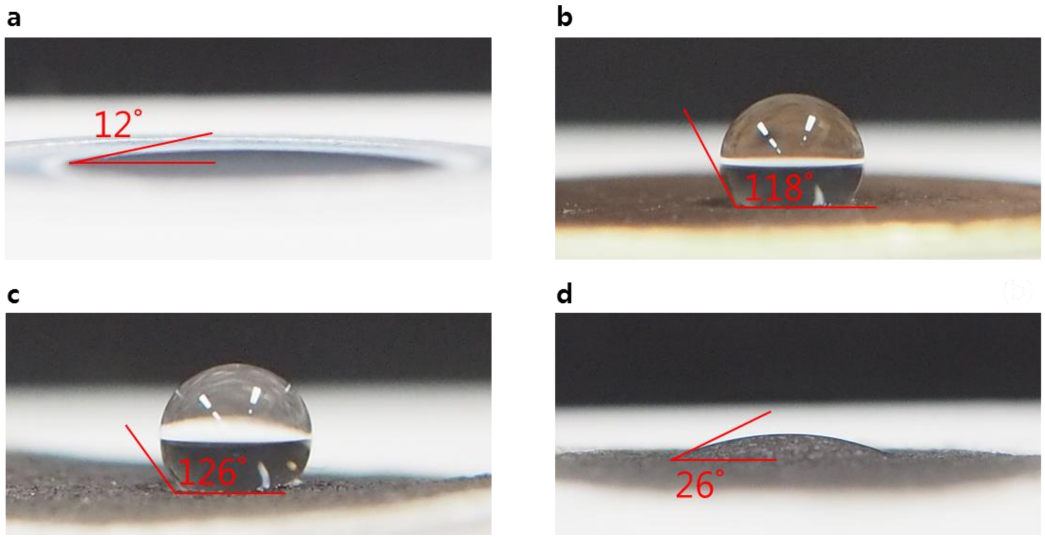

Supplementary Figure 7 | Contact angle of alumina nanowires and black gold membranes. Photograph of (**a**) self-aggregated alumina nanowires and the (**b**) black gold membrane before peeling off. (**c**) The membrane peeled off by 3M Micropore Surgical tape and (**d**) after soaking into the water. After soaking, the membrane turns into the hydrophilic surface which is more suitable for vapour generation.

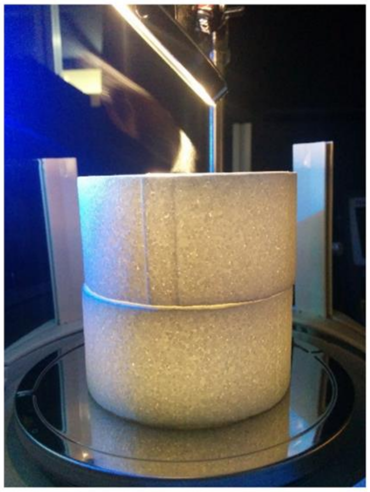

Supplementary Figure 8 | Photograph of vapour generation by black gold membrane.

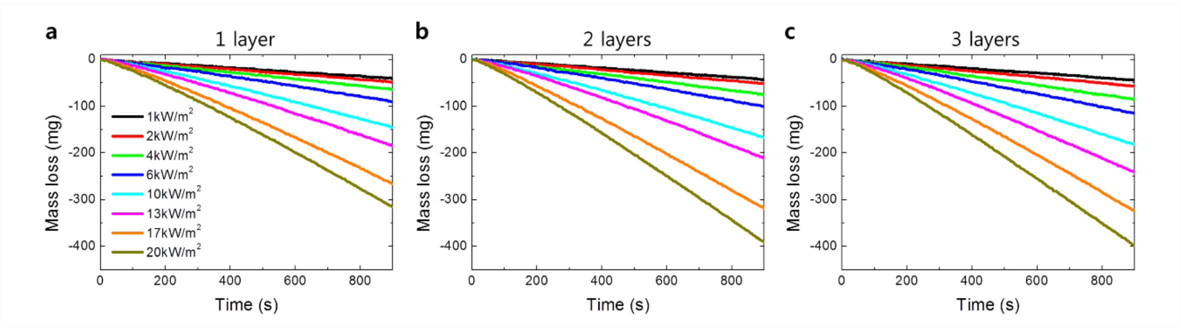

Supplementary Figure 9 | Mass change in various solar irradiations. The evaporation mass loss of water with different black gold membrane layers as (**a**) 1layer, (**b**) 2layers, and (**c**) 3layers.

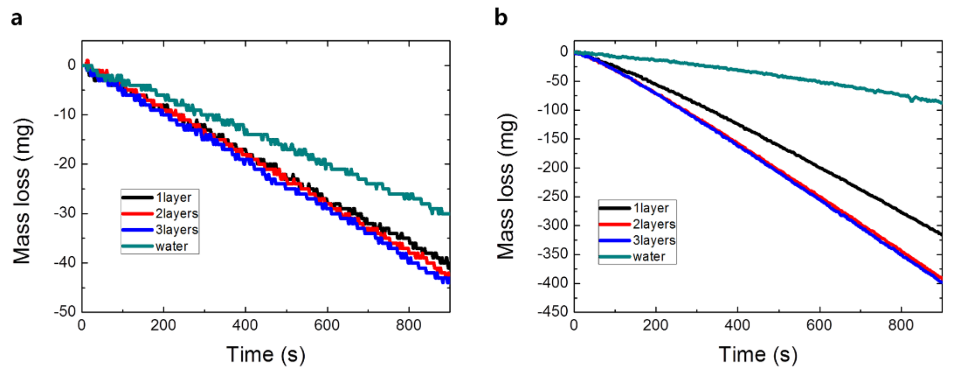

Supplementary Figure 10 | Evaporation under solar illuminations. The evaporation mass loss of water with various layers and without black gold membrane at (a) 1 kW m^{-2} and (b) 20 kW m^{-2}.

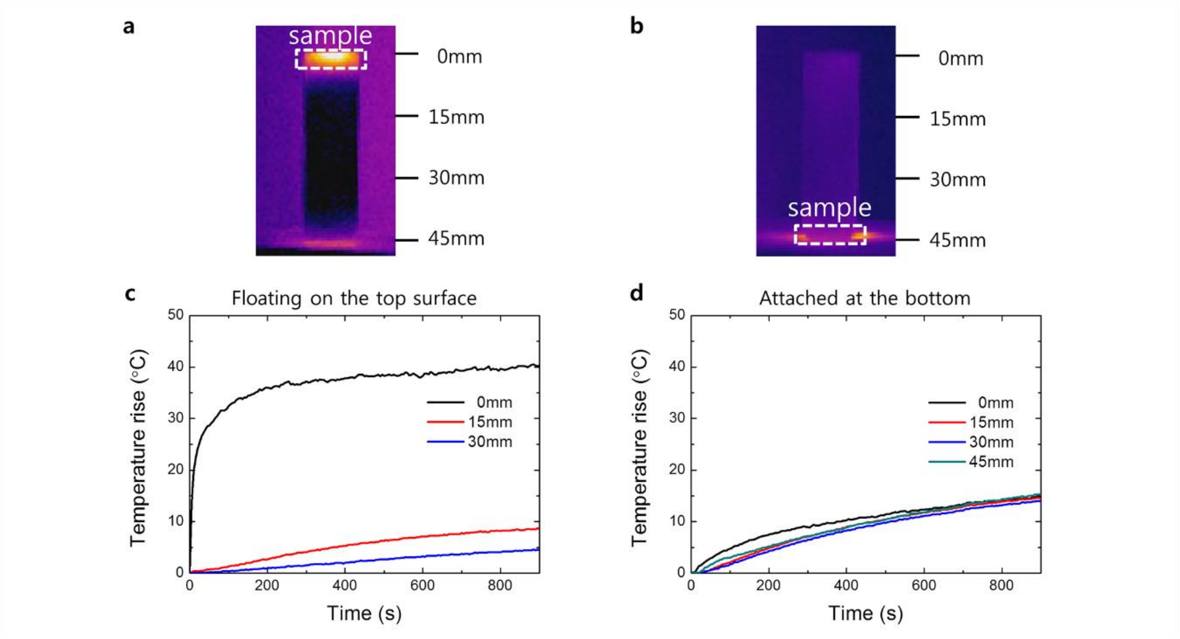

Supplementary Figure 11 | Temperature rises from floating and attached membrane. The black gold membrane floating on the top surface of the water and attached at the bottom of the cuvette. (**a-b**) IR images and (**c-d**) temperature rises, respectively.

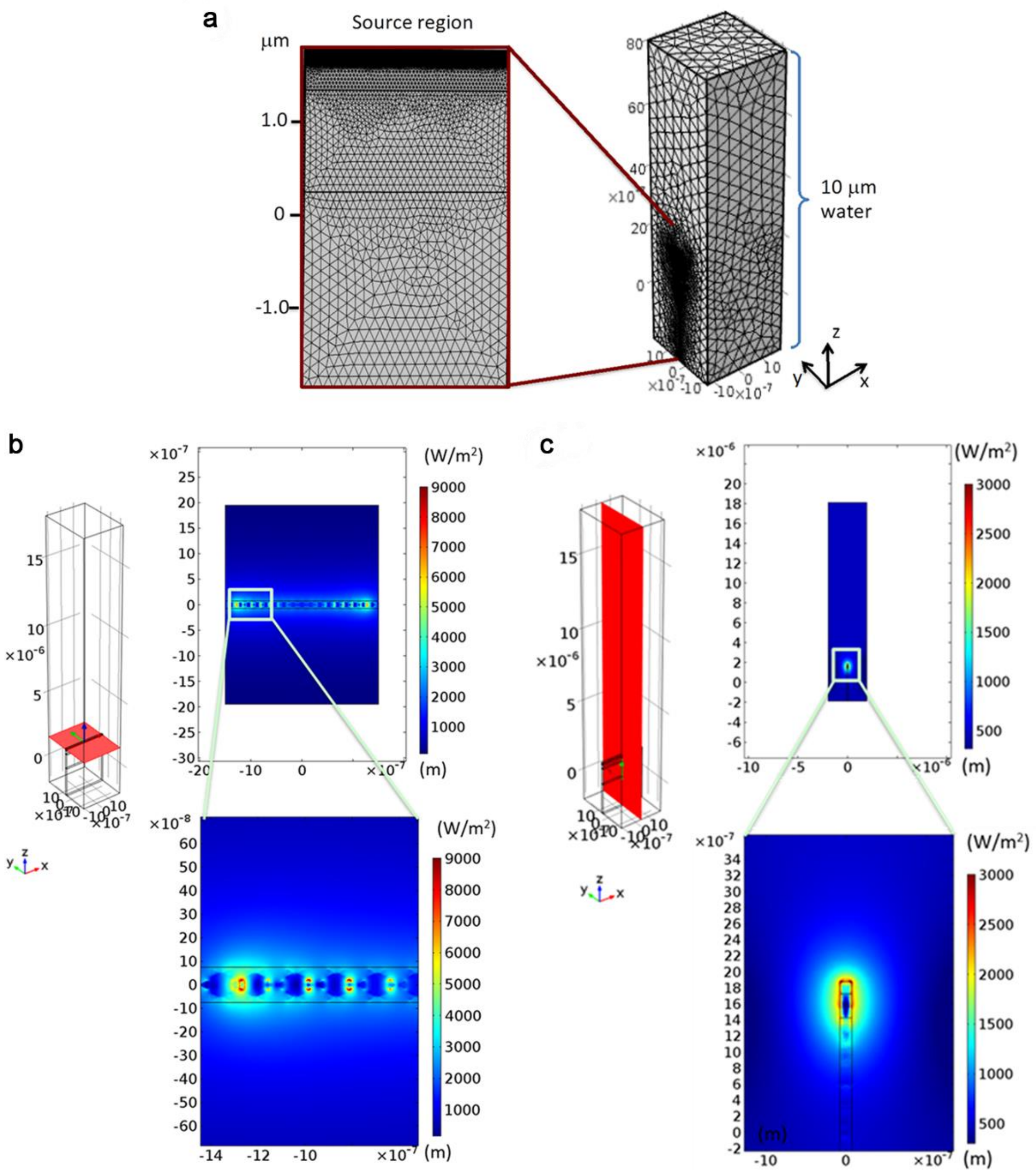

Supplementary Figure 12 | Thermal modeling. (**a**) Computational cell and meshing information. (**b**) A horizontal (xy) cross section of steady state heat flux profiles under 20 kW m^{-2} illumination. (**c**) A vertical (yz) cross section of steady state heat flux profiles under 20 kW m^{-2} illumination.

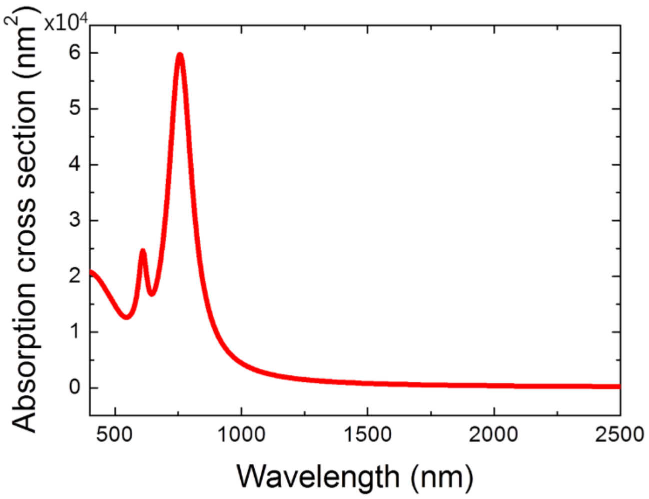

Supplementary Figure 13 | Absorption cross section of Au nanoshell.

Absorption cross section of the Au nanoshell that we used to compare with our black gold membrane for thermal simulations.
